# CXCR4-targeted theranostics in oncology

**DOI:** 10.1007/s00259-022-05849-y

**Published:** 2022-06-08

**Authors:** Andreas K. Buck, Sebastian E. Serfling, Thomas Lindner, Heribert Hänscheid, Andreas Schirbel, Stefanie Hahner, Martin Fassnacht, Hermann Einsele, Rudolf A. Werner

**Affiliations:** 1 Department of Nuclear Medicine, University Hospital Würzburg, Oberdürrbacher Str. 6, 97080 Wurzburg, Germany; 2 Division of Endocrinology and Diabetes, Department of Medicine I, University Hospital, University of Würzburg, Wurzburg, Germany; 3 Department of Internal Medicine II, Hematology and Oncology, University Hospital Würzburg, Wurzburg, Germany; 4 Division of Nuclear Medicine and Molecular Imaging, The Russell H Morgan Department of Radiology and Radiological Sciences, The Johns Hopkins School of Medicine, Baltimore, MD USA

**Keywords:** CXCR4, Theranostics, C-X-C motif chemokine receptor 4, [^68^Ga]PentixaFor, [^177^Lu]PentixaTher, [^90^Y]PentixaTher, Endoradiotherapy, Adrenocortical carcinoma, Multiple myeloma

## Abstract

A growing body of literature reports on the upregulation of C-X-C motif chemokine receptor 4 (CXCR4) in a variety of cancer entities, rendering this receptor as suitable target for molecular imaging and endoradiotherapy in a theranostic setting. For instance, the CXCR4-targeting positron emission tomography (PET) agent [^68^ Ga]PentixaFor has been proven useful for a comprehensive assessment of the current status quo of solid tumors, including adrenocortical carcinoma or small-cell lung cancer. In addition, [^68^ Ga]PentixaFor has also provided an excellent readout for hematological malignancies, such as multiple myeloma, marginal zone lymphoma, or mantle cell lymphoma. PET-based quantification of the CXCR4 capacities in vivo allows for selecting candidates that would be suitable for treatment using the theranostic equivalent [^177^Lu]/[^90^Y]PentixaTher. This CXCR4-directed theranostic concept has been used as a conditioning regimen prior to hematopoietic stem cell transplantation and to achieve sufficient anti-lymphoma/-tumor activity in particular for malignant tissues that are highly sensitive to radiation, such as the hematological system. Increasing the safety margin, pretherapeutic dosimetry is routinely performed to determine the optimal activity to enhance therapeutic efficacy and to reduce off-target adverse events. The present review will provide an overview of current applications for CXCR4-directed molecular imaging and will introduce the CXCR4-targeted theranostic concept for advanced hematological malignancies.

## Introduction

**Fig. 1:**
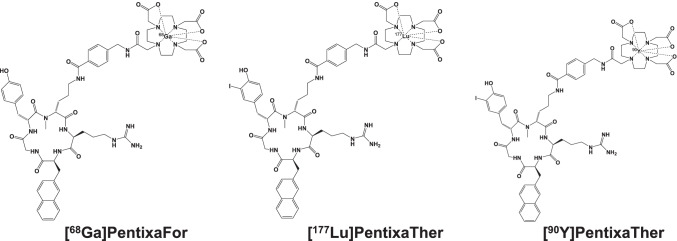
Chemical structures of [^68^Ga]PentixaFor, [^90^Y], and [.^177^Lu]PentixaTher

The C-X-C motif chemokine receptor 4 (CXCR4) has been recognized as a potential target for various applications in oncology and moderates crucial factors for cancer spread, such as angiogenesis or further involvement leading to therapeutic resistance [1]. Of note, ex vivo work-up revealed a large variety of solid cancers and hematological malignancies, which upregulate CXCR4 on the tumor cell surface, thereby rendering this G-protein coupled receptor as an attractive target for imaging and treatment [1]. Given its ability to precisely reflect sites of disease on a functional level, CXCR4-targeting radiotracers for single-photon emission computed tomography and positron emission tomography (PET) have been introduced for clinical use [2–6]. For instance, [^68^ Ga]PentixaFor has been extensively applied to patients affected with various solid and hematological neoplasms [7–10]. Radiotracer accumulation did not only reveal substantial correlation with immunohistochemical ex-vivo CXCR4 expression derived from corresponding tissue specimens [8], but was also more accurate in detecting metastatic sites, e.g., when compared to the current diagnostic work-up and standard imaging modalities in selected cases [11]. Of note, once [^68^Ga]PentixaFor has revealed substantial CXCR4 expression in vivo, the theranostic analogs [^177^Lu]/[^90^Y]PentixaTher can also be administered (Fig. 1) [12]. As such, CXCR4-directed PET also serves as a “one-stop” solution to determine the current status of disease spread and to identify patients eligible for a CXCR4-directed endoradiotherapy (ERT) using ß-emitters [13, 14]. In this regard, such treatment strategies have led to relevant anti-lymphoma/-tumor effect in selected cases and served as a conditioning regimen to enable for hematopoietic stem cell transplantation (HSCT) [12, 15]. Over the last decades, the theranostic concept has been primarily used and established in the clinic for treating solid tumors, such as prostate cancer or neuroendocrine neoplasms (NEN) [16, 17]. CXCR4-directed [^68^Ga]PentixaFor and [^177^Lu]/[^90^Y]PentixaTher, however, meet the urgent need to provide this innovative treatment strategy to patients affected with advanced blood cancer. In the present review, we will provide an overview of CXCR4-directed molecular imaging for solid tumors and hematologic malignancies. We will also review current therapeutic applications for hematological malignancies, including pretherapeutic dosimetry.

## CXCR4-directed molecular imaging

### Solid cancers

**Fig. 2:**
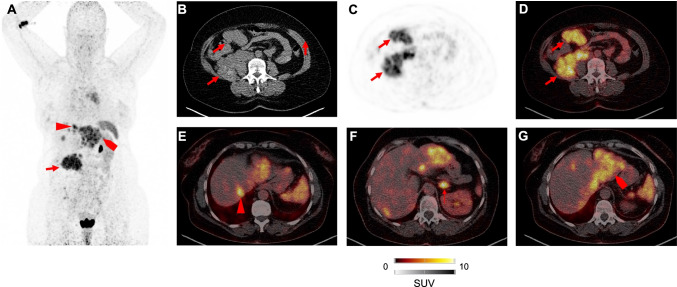
Patient after resection of adrenocortical carcinoma imaged with [^68^ Ga]PentixaFor. The right-sided primary was resected earlier. Maximum intensity projection in **A** revealed multiple sites of disease after administration of [^68^Ga]PentixaFor. Transaxial CT (**B**), PET (**C**), and PET/CT (**D**) demonstrated intense uptake in a retroperitoneal lesion. Further CXCR4 positive sites of disease included liver lesions (**E** and **G**). Additional discernible uptake in the left adrenal gland (**F**)

CXCR4-targeted PET has been first applied to patients diagnosed with solid tumors. Vag and co-workers included 22 patients with pancreatic cancer, prostate carcinoma, small-cell lung cancer (SCLC), melanoma, breast cancer, liver carcinoma, cancer of unknown primary, and glioblastoma, reporting on an increased radiotracer accumulation with high tumor-to-background ratio in SCLC [9]. In 10 patients, in whom 2-deoxy-2-[^18^F]fluoro-D-glucose ([^18^F]FDG) PET was available, the latter radiotracer exhibited higher standardized uptake values [9]. Another cohort of treatment-naïve patients affected with various solid cancers (including cholangiocarcinoma, ovarian cancer, and renal cell carcinoma) provided substantial correlation of tumor-derived specimens (defined as CXCR4-based immunoreactive scores) and [^68^ Ga]PentixaFor accumulation in sites of disease [8]. Among those cancer entities, cholangiocarcinoma had the highest uptake, which was up to sevenfold higher when compared to background [8]. In addition, work-up of tissue samples derived from patients with neuroendocrine neoplasms demonstrated that an increased proliferation index is linked to downregulation of somatostatin receptor (SSTR) 2 and 5, but upregulation of CXCR4 [18]. Those ex vivo findings were then further corroborated using SSTR-directed and [^68^ Ga]PentixaFor PET. In this regard, an increasing number of CXCR4( +)/SSTR( −) metastases were identified in patients with increasing tumor aggressiveness [19]. Previous studies, however, have already reported on the usefulness of [^18^F]FDG PET in the context of highly malignant, dedifferentiated neuroendocrine tumors [20, 21]. A retrospective head-to-head comparison of the latter radiotracer with [^68^Ga]PentixaFor PET demonstrated equal or inferior diagnostic performance with CXCR4 molecular imaging [22]. Nonetheless, given the rather limited treatment options for neuroendocrine tumor patients with a high proliferation index, [^68^Ga]PentixaFor may still allow to select potential treatment candidates for [^177^Lu] or [^90^Y]PentixaTher. In this regard, bone marrow ablation as a side effect would definitely occur and, thus, stem cell support would be needed [23]. Based on preliminary findings of Vag and coworkers [9] and promising ex vivo findings in lung cancer samples [24], Lapa et al. further investigated [^68^Ga]PentixaFor for treatment-naïve and pretreated SCLC and large-cell neuroendocrine carcinoma of the lung. In a comparison with SSTR-PET, the authors reported on an increased in-vivo CXCR4 expression [25]. A recent preclinical study also demonstrated increased ex-vivo CXCR4 expression in tissue specimens of patients affected with adrenocortical carcinoma (ACC) [26]. As an orphan disease, ACC has a less favorable prognosis in the vast majority of patients and, thus, novel treatment options are urgently needed [27, 28]. Bluemel and coworkers therefore investigated the read-out capabilities of [^68^Ga]PentixaFor in those patients. Although no substantial differences relative to [^18^F]FDG could be established in a visual and quantitative assessment, a markedly high number of subjects (70%) were rendered suitable for ERT using the theranostic counterparts [^177^Lu] or [^90^Y]PentixaTher [29]. In malignant pleural mesothelioma, human tissue samples also revealed robust CXCR4 expression in an ex-vivo setting [30], which then again provided a rationale to investigate [^68^Ga]PentixaFor in this disease [30]. Of note, ex-vivo findings were not confirmed by an in-vivo molecular imaging approach, as no substantial radiotracer accumulation was recorded [31], which further demonstrates that an ex-vivo proof of CXCR4 expression does not always lead to increased uptake on PET. Taken together, SCLC, cholangiocarcinoma, highly dedifferentiated NEN, and ACC may be the most promising tumor entities for a CXCR4-directed PET (Fig. 2) [8, 19, 25, 29].

A recent study comprised more than 145 solid tumor patients focusing on a potential predictive role of physiological splenic uptake and outcome [32]. In lung carcinoma and NEN, the authors reported on a substantial interrelation between thrombocytes and white blood cell counts and radiotracer accumulation in the spleen as a hematopoetic reservoir involved in the immune response. As such, further studies are needed to elucidate the role of systemic inflammation detected by CXCR4 PET in those tumor subtypes [32]. Another recently published study investigated a potential tumor sink effect in the context of CXCR4-directed PET [33], as such a decrease of uptake in normal organs in subjects with increased tumor load has been reported for other theranostic agents, e.g., somatostatin receptor directed radiopharmaceuticals [34]. If such a tumor sink effect also occurs in patients injected with [^68^Ga]PentixaFor, this may have a relevant impact on “hot” and “cold” therapies targeting CXCR4, e.g., by safely increasing the amount of therapeutic activity but reducing side effects in organs with normal biodistribution [33]. Investigating this effect on [^68^ Ga]PentixaFor in 90 patients with solid tumors, the authors did not report on decreasing radiotracer accumulation in patients with higher tumor burden, further supporting the hypothesis that doses in normal organs and sites of disease can rather not be estimated based on pretherapeutic PET. In this regard, those findings favor the use of treatment planning using dosimetry [33].

### Advanced hematological malignancies

Multiple studies demonstrated that [^68^Ga]PentixaFor may be particularly useful for imaging various types of advanced blood cancers. As such, the first biodistribution study for this radiotracer was conducted in 5 subjects diagnosed with multiple myeloma (MM) and reported on an effective of 2.3 mSv [3], which was comparable to other 68-Ga-labeled theranostic radiotracers [35]. MM has also been further investigated with [^68^ Ga]PentixaFor [36], demonstrating remarkable diagnostic accuracy for identifying MM manifestations, which was superior relative to [^18^F]FDG in newly diagnosed subjects (positive rate almost twice for CXCR4 PET) [37]. Further demonstrating a tight interaction with disease state and in-vivo CXCR4 expression, uptake in bone marrow was associated with staging or relevant markers of disease activity, e.g., serum-free light chain or ß2-microglobulin [37]. Of note, the derived PET signal may also hold potential for outcome prediction, as a negative scan was linked to increased time-to-progression and overall survival [7]. Among hematological malignancies, [^68^Ga]PentixaFor has also been first applied to patients affected with acute myeloid leukemia in a translational setup. Using flow cytometry, increased patient-derived high blast counts were linked to CXCR4 upregulation. In mice affected with either CXCR4( −) or CXCR4( +) leukemia xenografts, an increased [^68^Ga]PentixaFor signal was observed in the latter animals. Last, in 10 patients with active disease, elevated radiotracer uptake was tightly linked to disease infiltration by magnetic resonance in half of the investigated subjects [38].

**Fig. 3:**
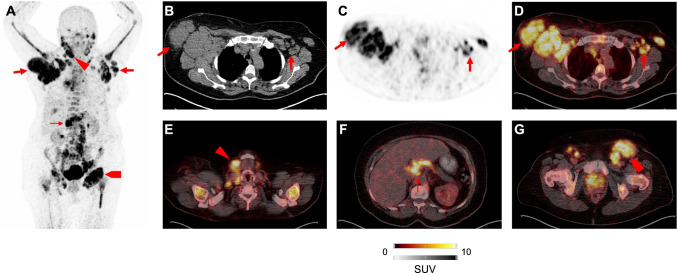
Patient with marginal zone lymphoma after injection of [^68^ Ga]PentixaFor. Multiple disease sites are visualized on maximum intensity projection in **A**. Transaxial CT (**B**), PET (**C**), and PET/CT (**D**) revealed intense lymph node manifestations in the thorax. PET/CT also showed radiotracer accumulation in the cervical (**E**), abdominal (**F**), and in the inguinal region (**G**). Modified from Duell et al., Journal of Nuclear Medicine, October 2021, 62 (10) 1415–1421 [11]. © by the Society of Nuclear Medicine and Molecular Imaging, Inc

Moreover, CXCR4-directed imaging has also been applied to 22 treatment-naïve patients affected with marginal zone lymphoma (MZL) [11]. When compared to routine clinical work-up (including endoscopy of the gastrointestinal tract and bone marrow biopsy), [^68^Ga]PentixaFor PET, but not standard procedures, classified all cases correctly [11]. Of interest, PET changed both staging and therapeutic management, further indicating that this radiopharmaceutical could be applied to routine assessment in individuals affected with MZL (Fig. 3) [11]. Specimen of gastric mucosa-associated lymphoid tissue (MALT) lymphoma also revealed CXCR4 overexpression in an ex-vivo setup [39] and those findings were further corroborated in an in-vivo setting, demonstrating an accuracy of 100% (with gastric biopsies serving as reference) in subjects after *Helicobacter pylori* eradication [10], thereby demonstrating that this radiotracer can assess residual disease activity [10]. The same research group also investigated [^68^Ga]PentixaFor PET for mantle cell lymphoma (MCL), as the diagnostic performance of the currently applied radiotracer [^18^F]FDG is hampered by increased uptake in the bone marrow [40]. Relative to the latter radiotracer, the CXCR4 agent demonstrated an increased sensitivity of up to 25% on a per region level [40]. A quantitative assessment also demonstrated higher target-to-background ratios, rendering [^68^Ga]PentixaFor as a suitable alternative to [^18^F]FDG in MCL [40]. CXCR4-directed PET was also used in myeloproliferative neoplasms (including essential thrombocythemia and polycythemia vera) and all of the included 12 patients revealed positive findings [41]. Further corroborating the clinical relevance, the SUV reduction of a baseline and follow-up [^68^Ga]PentixaFor scan correlated with decrease of spleen volume, supporting the hypothesis that quantitative parameters may be also applicable for response assessment [41]. As a relatively rare form of non-Hodgkin lymphoma, utility of [^18^F]FDG is also limited in Waldenström macroglobulinemia/lymphoplasmacytic lymphoma (again, due to bone marrow involvement leading to rather less specific uptake) [42]. Luo et al. reported on a substantial higher rate of positive findings after injection of [^68^Ga]PentixaFor when compared to [^18^F]FDG [42].

### Roadmap of relevant in-vivo CXCR4 expression

**Fig. 4:**
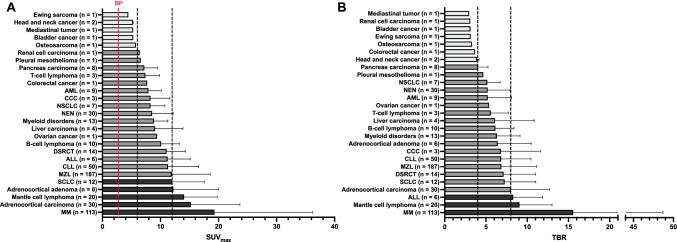
Bar chart showing **A** average SUV_max_ and **B** target-to-background ratio (TBR). For **A**, black dotted lines indicate SUV_max_ cutoffs of 6 and 12, and for **B**, those lines show TBR cutoffs of 4 and 8, respectively. *BP* blood pool (red dotted line), *AML* acute myeloid leukemia, *CCC* cholangiocarcinoma, *NSCLC* non-small-cell lung carcinoma, *NEN* neuroendocrine neoplasm, *DSRCT* Desmoplastic Small Round Cell Tumor, *ALL* acute lymphoblastoid leukemia, *CLL* chronic lymphocytic leukemia, *MZL* marginal zone lymphoma, *SCLC* small-cell lung carcinoma, *MM* multiple myeloma. Adrenocortical adenoma: aldosteron-producing adrenocortical adenoma. Modified from Buck et al., Journal of Nuclear Medicine, 2022 Mar 3; jnumed.121.263693 [43]. © by the Society of Nuclear Medicine and Molecular Imaging, Inc

Aiming to provide a roadmap among a broad spectrum of neoplasms, a recent bicentric study of our group and colleagues from Vienna Medical University assessed [^68^Ga]PentixaFor uptake and image contrast among the largest cohort of subjects imaged with CXCR4-directed PET to date, thereby determining the most relevant clinical applications. Investigating 690 patients affected with various solid tumors and hematological neoplasms scheduled for 777 scans, 68.9% demonstrated uptake in sites of disease [43]. The highest tracer uptake was recorded in MM (maximum SUV > 12). The second highest uptake was then found in ACC, MCL, adrenocortical adenoma, and SCL. Osteosarcoma, bladder cancer, head and neck cancer, and Ewing sarcoma, on the other hand, exhibited the lowest average SUV (< 6; Fig. 4A) [43]. Comparable findings were recorded for target-to-background ratio (TBR), thereby reflecting image contrast. Again, the highest TBR was found in advanced blood cancers, including MM, MCL, and acute lymphoblastoid leukemia (Fig. 4B) [43]. Moreover, lower specific activity is characterized by higher amounts of cold mass, thereby having a relevant impact on image interpretation [44]. The authors did not record any relevant significant associations with semiquantitative parameters and specific activity, supporting the hypothesis that read-out capabilities are not hampered, regardless of the amount of specific activities [43].

## CXCR4-targeted endoradiotherapy

### Biokinetics and pretherapeutic dosimetry

**Fig. 5:**
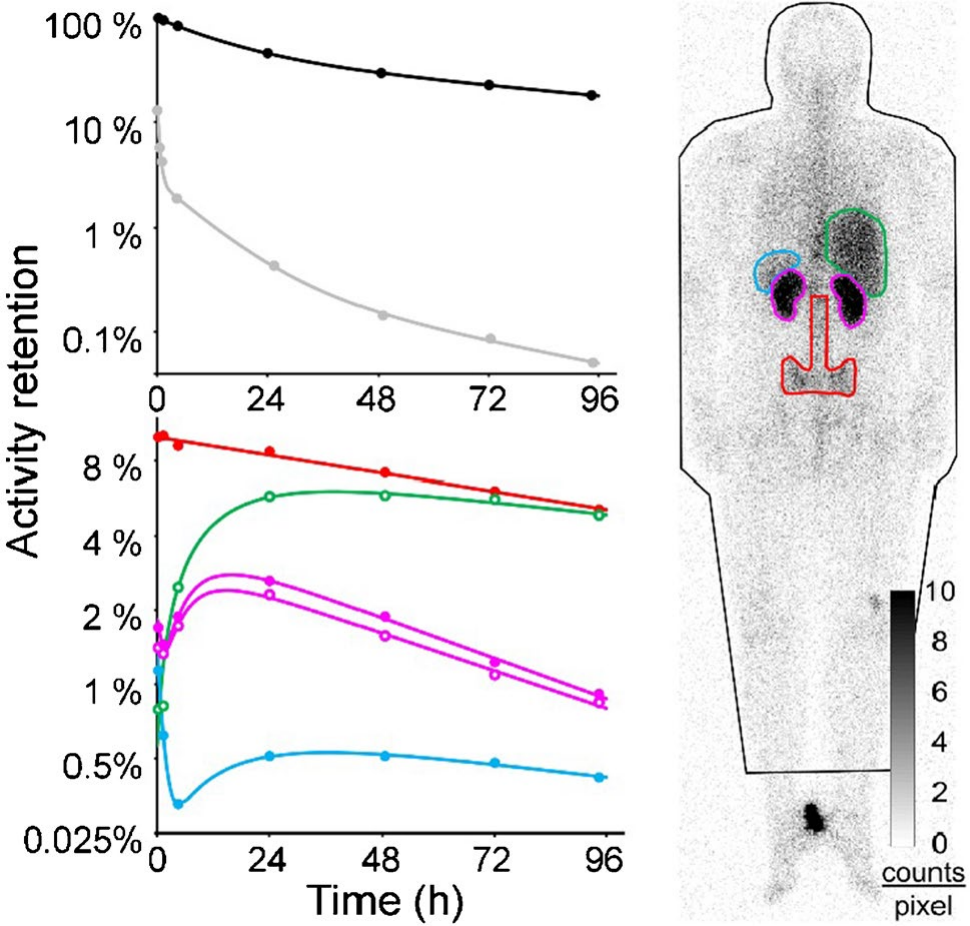
Example of activity time functions in a patient with multiple myeloma. Activity retention measurements as well as fit functions are shown for the whole body (black), per liter of whole blood (grey), red bone marrow (red), liver (green), kidneys (purple), and spleen (blue). Modified from Hänscheid et al., Journal of Nuclear Medicine 2021 Aug 19; jnumed.121.262295, https://doi.org/10.2967/jnumed.121.262295 [46]. © by the Society of Nuclear Medicine and Molecular Imaging, Inc

After intravenous administration, [^177^Lu]PentixaTher binds to plasma proteins with high metabolic stability, and only a small fraction of about 4% is attached to leukocytes and platelets via CXCR4 binding [45]. Scintigraphically detectable activity accumulations are found in kidney, liver, spleen, and bone marrow, as well as in CXCR4-expressing malignant tissues. An example of measured time functions of activity retention in organs and tissues in a patient with MM is shown in Fig. 5. The figure, like the results summarized below unless otherwise specified, is taken from a recently published study on [^177^Lu]PentixaTher biokinetics and dosimetry [46].

The total body ^177^Lu activity typically decays bi-exponentially. About half of the activity is eliminated with a median effective half-life of about 10 h mainly by renal excretion; the remainder decays with a mean effective half-life of about 4 days. Activity concentration in blood typically shows three components with about 10%, 2.5%, and 0.2% of the administered activity per liter of blood decaying with half-lives of 0.23 h, 7 h, and 40 h, respectively.

[^177^Lu]PentixaTher accumulates in the bone marrow and remains there with a half-life of several days, making the bone marrow the critical organ where acute toxicity is foremost expected. The calculated specific bone marrow doses were heterogeneous, ranging from 0.14 to 2.3 (median value, 0.5) Gy/GBq ^177^Lu. Given high individual variability and the uncertainties of bone marrow dosimetry, therapeutic use of PentixaTher may be confined to myeloablative therapies. However, it must be considered in myeloablative treatment that the long residence time of the activity in the bone marrow requires a long decay time before a stem cell transplantation can be safely performed. Therefore, in order to reduce the duration of the phase of aplasia and the associated risk of threatening complications, therapy is usually performed with the nuclide ^90^Y instead of ^177^Lu [46].

In myeloablative treatment, therapeutic activity is limited by the absorbed dose to the kidneys. As with other radiolabelled peptides such as [^177^Lu]DOTA-TOC/TATE [47], a fraction of the active compound filtered by the kidneys is retained in renal tubules, leading to an initial increase of the retention per kidney up to a mean maximum uptake of 2.2% of the administered [^177^Lu]PentixaTher activity, approximately 18 h after administration [46]. The mean effective half-life of the activity elimination from kidneys by degradation of the compound and physical decay is 41 ± 10 h [46]. In the kidneys as well, large heterogeneity of specific absorbed doses have been observed with values between 0.4 and 3.5 (median: 0.9) Gy/GBq ^177^Lu [46]. It has been reported that the accumulation of PenixaTher in the kidneys was reduced to 64% ± 13% by the concomitant administration of amino acids [48]; however, this value was determined using data from only six patients receiving [^177^Lu]PentixaTher, showing reduction factors ranging from 50 to 80% [48].

Liver and spleen show delayed kinetics compared to kidneys. Specific absorbed doses are often high but never restrict the administrable activity. High splenic doses are often observed and therapeutically desirable in patients with hematologic disease with malignant infiltration of the spleen, often associated with splenomegaly [46].

A long half-life of [^177^Lu]PentixaTher of 122 ± 32 h is also found in tumors and extramedullary lesions in hematological diseases. The absorbed doses are typically twice as high as in the critical organ, the kidneys. Since myeloablative therapy cannot be repeated several times, therapy with PentixaTher is primarily promising for tissues that are very sensitive to radiation, such as the hematological system [46].

In order to plan therapy with radioactively labeled PentixaTher and to estimate the activity that can be safely administered, the kinetics of at least kidneys and, if possible, the target tissue, should be measured. A sufficiently reliable pretherapeutic dosimetry is possible with 200 MBq [^177^Lu]PentixaTher [46]. Due to the short half-life in the kidneys, daily measurements over 4 days are usually sufficient for treatment with ^177^Lu and measurements over 3 days for therapy with ^90^Y. The relative time course of the activity in tissues of interest is determined by identically executed planar scans or SPECT. Bi-exponential functions are usually adequate for fitting activity-time functions to the measured count rates. At least one SPECT/CT with correction for attenuation and scatter in the reconstruction is required to assess absolute activity concentrations used to normalize the activity time functions [46, 47].

### Efficacy

**Fig. 6:**
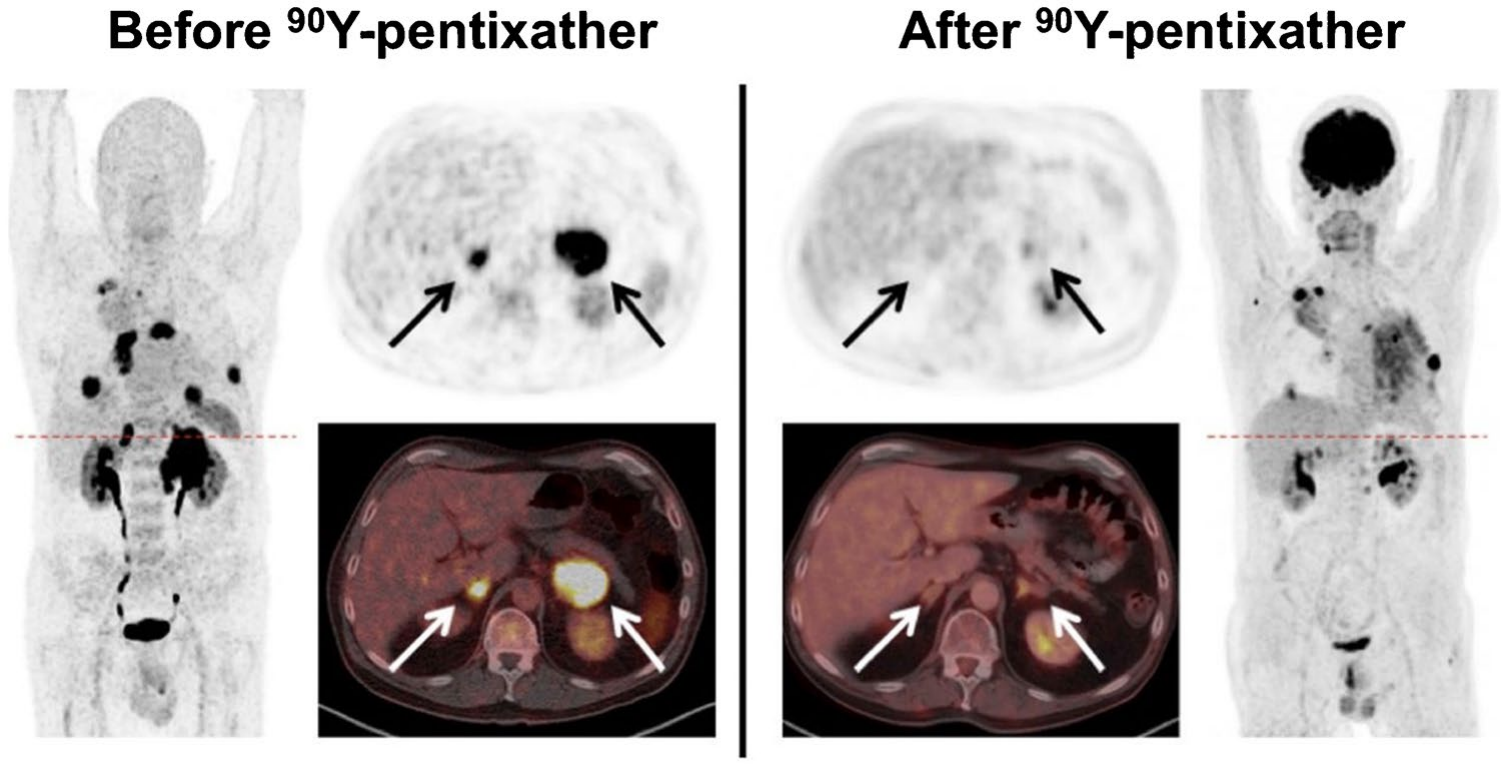
Partial response in a patient affected with diffuse large B cell lymphoma treated with CXCR4-targeted endoradiotherapy and additional radioimmunotherapy. Pretherapeutic [^68^Ga]PentixaFor (left) and posttherapeutic [^18^F]FDG PET/CT (right) after tandem treatment using [^90^Y]PentixaTher and [^90^Y]Ibritumomab-Tiuxetan (Zevalin). Posttherapy scans demonstrated reduction of lesions in the kidneys, adrenals (arrows), lung, and nodal disease manifestations. Note that diffuse radiotracer accumulation in the lung on [^18^F]FDG maximum intensity projection on the right was due to pneumonia. Modified from Lapa et al., Journal of Nuclear Medicine Jan 2019, 60 (1) 60–64 [15], © by the Society of Nuclear Medicine and Molecular Imaging, Inc

After having visualized CXCR4 expression of tumor lesions in an in vivo setting, patients can be scheduled for CXCR4-directed ERT using the ß-emitting theranostic twin [^177^Lu]/[^90^Y]PentixaTher (Fig. 1). Using human cell lines and a tumor-bearing murine lymphoma model, Schottelius et al. reported on increased radiotracer accumulation over time in tumor sites [45]. In a translational approach, those preclinical investigations paved the way for the injection of [^177^Lu]PentixaTher in a patient affected with MM, leading to successful bone marrow ablation [45]. Herrmann and coworkers also reported on three MM patients, which underwent CXCR4-directed ERT, followed by chemotherapy and autologous HSCT [48]. During follow-up, a remarkable response was noted with two patients achieving either partial (PMR) or complete metabolic response (CMR) [48]. Based on these promising results, another study reported on 8 advanced, extensively pretreated MM patients scheduled for CXCR4 ERT, also reporting on PMR and CMR in 6/8 cases [49]. Despite such remarkable anti-myeloma activity, one subject succumbed to sepsis and another patient with extremely high tumor burden to tumor lysis syndrome [49]. Further increasing the safety margin, protocols to prevent such syndromes can be employed, preferably starting prior to on-set of CXCR4 ERT [50]. The same group also reported on CXCR4-targeted ERT in acute lymphoblastic and myeloid leukemia. After having assessed the target capacities in vivo by PET, PentixaTher was administered to three subjects with refractory disease. After successful myelosuppression, all patients underwent allogeneic hematopoietic stem cell transplantation, thereby paving the way for successful engraftment [12]. The concept of CXCR4 ERT has also been applied to patients affected with diffuse large B cell lymphoma, which were treated with [^90^Y]PentixaTher in combination with CD20/CD66 radioimmunotherapy, also followed by chemotherapy and allogeneic HSCT [15]. In patients treated with combined [^90^Y]PentixaTher ERT and radioimmunotherapy, PR was achieved (Fig. 6) [15].

### Toxicity profile

**Fig. 7:**
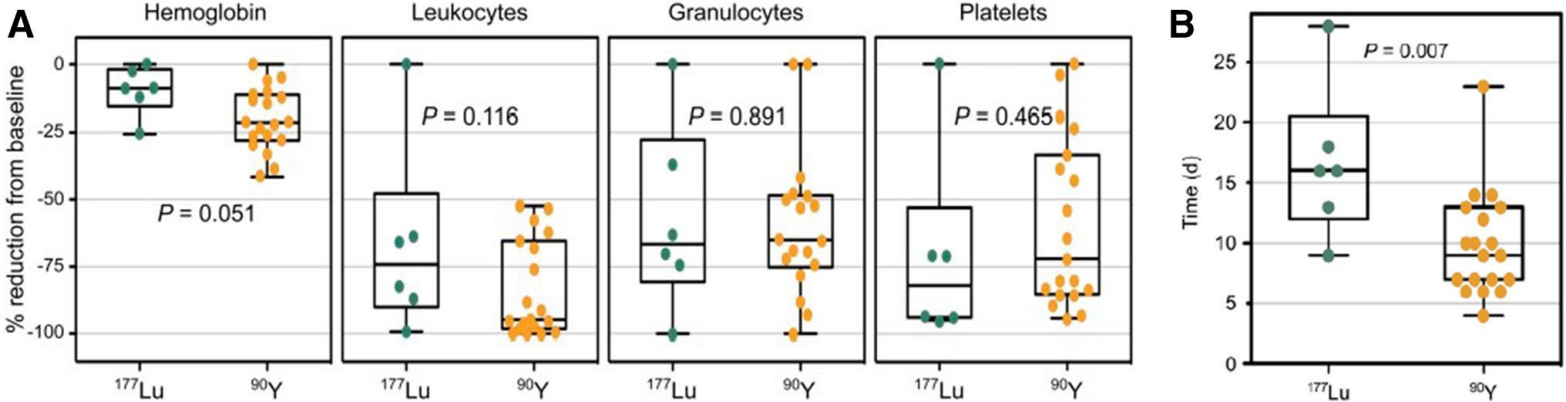
**A** Reduction of blood values relative to baseline after CXCR4-directed endoradiotherapy. **B** Time interval between CXCR4-directed endoradiotherapy and start of conventional conditioning therapy. Desired cytopenia was achieved for both [^90^Y]PentixaTher and [^177^Lu]PentixaTher (**A**). For [^90^Y]PentixaTher, however, the time interval until start of conditioning therapy was significantly shorter, which can be explained by the longer half-life of 2.7 days (^177^Lu, 6.7 days) (**B**). Modified from Maurer et al., 2019 Oct; 60(10): 1399–1405 [23], © by the Society of Nuclear Medicine and Molecular Imaging, Inc

Investigating the safety profile, 22 patients with advanced blood cancer treated with [^177^Lu] or [^90^Y]PentixaTher and subsequent chemotherapy followed by HSCT were investigated [23]. As expected, all patients developed cytopenia (including hemoglobin, leukocytes, granulocytes, and platelets; Fig. 7A) [23]. One patient developed tumor lysis syndrome, followed by grade 3 acute kidney failure, while all other adverse effects were manageable and did not cause any delay for further treatment [23]. In this regard, time interval between CXCR4 ERT and conditioning therapy was significantly longer with [^177^Lu]PentixaTher, which can be explained by the longer half-life of 6.7 days when compared to [^90^Y]PentixaTher (2.7 days; Fig. 7B) [23]. The ongoing COLPRIT trial is a prospective phase I/II study which will further elucidate the therapeutic efficacy and safety of this theranostic strategy in patients with advanced blood cancer (Eudra‐CT 2015‐001817‐28).

Table 1 provides an overview of conducted CXCR4 therapies to date, including maximum achieved tumor doses and responses.

**Table 1:** Overview of CXCR4-directed endoradiotherapies in advanced hematological malignancies

*Study*	*Type of blood cancer*	*No. of patients*	*Used radionuclide*	*Administered activity (GBq)*	*Achieved Gy to tumor sites (maximum)*	*Outcome*	
						SAE	Best response
*Herrmann *et al*. *[48]	MM	3	[^177^Lu]/[^90^Y]PentixaTher	6.3–23.5	84	Death (1/3, sepsis)	PR (1/3), CMR (1/3)
*Habringer *et al*. *[12]	AML	3	[^90^Y[PentixaTher	2.7–4.72	53	Death (1/3, sepsis)	CMR (1/3), NA in 1/3
*Lapa *et al*. *[49]	MM	8*	[^177^Lu]/[^90^Y]PentixaTher	2.6–23.5	> 70	2/8 death (sepsis, lethal TLS)	5/8 PR, 1/8 CMR
*Lapa *et al*. *[15]	DLBCL	6	[^90^Y[PentixaTher	2.8–6.5	96.5	Death (2/6, sepsis and CNS aspergillosis)	2/6 PR^§^, MR in 2/6
*Maurer *et al*. *[23]	AML, MM, DLBCL, MCL, T-PLL	22	[^177^Lu]/[^90^Y]PentixaTher^#^	7.6–23.5	NA	TLS with grade 3 kidney failure (1/22)	NA (investigation of side effects)

*SAE* severe adverse event, *MM* multiple myeloma, *PR* partial response, *CMR* complete metabolic response, *AML* acute myeloid leukemia, *NA* not available, *TLS* tumor lysis syndrome, *DLBCL* diffuse large B cell lymphoma, *CNS* central nervous system, *MR* mixed response, *MCL* mantle cell lymphoma, *T-PLL* T-cell prolymphocytic leukemia

*One patient treated with three cycles

^§^Treated with additional radioimmunotherapy

^#^8/22 treated with additional radioimmunotherapy

## Future aspects

### Expanding CXCR4-targeted theranostics to solid tumors

To date, [^177^Lu]/[^90^Y]PentixaTher has been applied to patients affected with various types of blood cancers [12, 15, 48, 49], not only to achieve an anti-tumor effect, but also as a conditioning regimen followed by allogenic or autologous HSCT. Although such a stem cell backup is mandatory due to bone marrow ablation, CXCR4-directed ERT could also be applied to solid tumor patients exhibiting increased CXCR4 expression on PET [43]. Such an approach, however, would be restricted to refractory end-stage disease patients having exhausted all other treatment lines. For instance, in ACC patients, treatment options are limited [51] and, thus, administration of [^90^Y]PentixaTher may be feasible as a salvage approach, e.g., after having harvested stem cells during previous chemotherapeutic protocols [52].

### Image-guided therapy for non-radioactive CXCR4-directed drugs

Despite treatment with hot CXCR4-directed radiotracers, [^68^Ga]PentixaFor could also be applied to patients scheduled for treatment with cold drugs also interacting with this chemokine receptor. Among others, those medications include small molecule (AMD3100/plerixafor), molecules targeting CXC12 (NOX-12, CX-01), peptide-based molecules (BL-8040, LYS2510924, POL5551), or antibodies (ulocuplumab). Such agents have been partially investigated in humans as chemosensitizing agents, e.g., for acute myeloid leukemia and ALL, but with rather disappointing results [53]. Pretherapeutic [^68^Ga]PentixaFor PET could assess the current status quo of the target and may provide guidance towards better patient selection. In addition, ex-vivo CXCR4 overexpression has been advocated to be tightly linked to worse prognosis in those patients, e.g., in ALL [54, 55] and CXCR4 may be also involved in chemotherapeutic resistance [56]. As such, in vivo molecular imaging may then also be useful to identify such high risks prone to chemotherapy failure or as a prognostic tool for further clinical outcome.

### Systemic networking on CXCR4-PET to assess cardiovascular toxicity as an adverse effect of anti-tumor treatment

A recent study enrolling oncology patients revealed increased in-vivo expression of fibroblast activation protein not only in metastases, but also in the myocardium [57]. Such a complex interplay between tumor and the cardiovascular system could also be assessed in future studies using [^68^Ga]PentixaFor. In this regard, numerous studies have already reported on the feasibility of CXCR4-directed PET in patients after myocardial infarction [58–60]. Cardio-oncology studies investigating interactions between the heart, vessels, and tumor sites may allow to detect subjects developing relevant off-target effects caused by their anti-tumor therapeutic regimen [61]. Such studies demonstrating a potential inflammatory activity in large arteries have already been conducted using a retrospective cohort of melanoma patients imaged with [^18^F]FDG and treated with immune checkpoint inhibitors, which are known to cause myocarditis and potential life-threating cardiovascular events [62, 63]. Relative to [^18^F]FDG, however, CXCR4 PET has already identified a higher number of atherosclerotic lesions in the vessel wall in oncology patients and, thus, may even provide a more reliable read-out of ongoing inflammatory activities under tumor-specific treatment [64].

## Conclusions

CXCR4 is upregulated on various cancer cells, rendering this receptor as a potential target for tumor read-out and treatment strategies [1]. The CXCR4-targeted PET agent [^68^Ga]PentixaFor has been successfully applied to patients with solid and advanced blood cancers, demonstrating substantially increased radiotracer accumulation in ACC, SCLC, MM, MZL, MCL, or gastric MALT [10, 11, 25, 29, 37, 40]. In addition to assessment of widespread disease, such a functional imaging approach allows to assess the capacities of the target in-vivo. Thus, quantification of [^68^Ga]PentixaFor accumulation may then allow to estimate the efficacy of non-radioactive CXCR4 inhibitory treatments (e.g., with anti-human CXCR4 IgG monoclonal antibodies for MM patients) [65] or to identify patients that would be eligible for treatment with hot CXCR4-directed theranostic radiotracers, such as [^177^Lu]/[^90^Y]PentixaTher [46]. The latter concept has already been applied to hematological malignancies known to be sensitive to radiation, e.g., in advanced MM, ALL, or diffuse large B cell lymphoma [12, 15, 49]. In this context, pretherapeutic dosimetry can determine the appropriate amount of activity to achieve anti-tumor effects and to minimize off-target effects [46]. CXCR4 ERT also caused desired bone marrow ablation and has therefore been incorporated in the therapeutic algorithm of advanced blood cancer patients (allogenic/autologous HSCT following CXCR4 ERT along with successful engraftment) [12, 15, 49]. Therapeutic efficacy of those treatment regimens led to remarkable outcome benefits in those heavily pretreated patients [12, 15, 23, 49]. Given substantial high doses in the tumor, some patients experienced tumor lysis syndrome and thus, those individuals should be closely monitored [23].
